# Product Peroxidation Inhibition in Methane Photooxidation into Methanol

**DOI:** 10.1002/advs.202306891

**Published:** 2024-01-17

**Authors:** Yuehan Cao, Zeai Huang, Chunqiu Han, Ying Zhou

**Affiliations:** ^1^ National Key Laboratory of Oil and Gas Reservoir Geology and Exploitation Southwest Petroleum University Chengdu 610500 China; ^2^ School of New Energy and Materials Southwest Petroleum University Chengdu 610500 China

**Keywords:** active site mechanism, inhibition of methanol peroxidation, methane photooxidation, radical mechanism

## Abstract

Methane photooxidation into methanol offers a practical approach for the generation of high‐value chemicals and the efficient storage of solar energy. However, the propensity for C─H bonds in the desired products to cleave more easily than those in methane molecules results in a continuous dehydrogenation process, inevitably leading to methanol peroxidation. Consequently, inhibiting methanol peroxidation is perceived as one of the most formidable challenges in the field of direct conversion of methane to methanol. This review offers a thorough overview of the typical mechanisms involved radical mechanism and active site mechanism and the regulatory methods employed to inhibit product peroxidation in methane photooxidation. Additionally, several perspectives on the future research direction of this crucial field are proposed.

## Graphical abstract

This review presents a comprehensive summary of the typical mechanisms employed to inhibit methanol peroxidation, including both the radical mechanism and the active site mechanism, and related various methods for modifying photocatalysts to effectively suppress methanol peroxidation during the methane photooxidation process are summarized. Additionally, several perspectives on the future research direction of this crucial field are proposed.

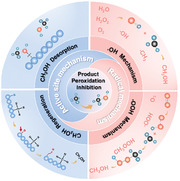

## 1 Introduction

Contrasted with oil (approximately CH) or coal (approximately CH_2_), methane (CH_4_), the primary constituent of natural gas, shale gas, and coalbed gas, etc., is viewed as the cleanest form of fossil energy due to its lower C/H ratio.^[^ ^1^ ^]^ Beyond its utilization as a fuel, methane is also recognized as an essential feedstock for C1 chemistry.^[^ ^2^ ^]^ Using methane more for chemical production (e.g., methanol) instead of exothermic combustion could maximize the value of methane and mitigate carbon emissions in the atmosphere.^[^ ^3^ ^]^ Taking methanol (CH_3_OH) as an example, its energy density is ≈17 MJ l^−1^, which is nearly 400 times higher than that of CH_4_.^[^ ^3^, ^4^ ^]^ Notably, CH_3_OH is extensively used as a raw material for producing various organic products in the fine chemicals industry (e.g., olefins, aldehydes, and organic acids, etc.).^[^ ^5^ ^]^ Furthermore, considering the diminishing reserves of crude oil and coal and the projected substantial reserves of methane hydrate, shale gas, etc. (attributing to the production of 4036.9 billion cubic meters of worldwide natural gas in 2022 as analyzed by the BP Statistical Review of World Energy), it becomes increasingly important to leverage methane as the building block for chemical synthesis in the approaching decades.^[^ ^6^ ^]^

In industry, an indirect method is typically used to convert CH_4_ into CH_3_OH.^[^ ^7^ ^]^ This process often involves the production of syngas (a mixture of carbon monoxide (CO) and hydrogen (H_2_)) through highly endothermic steam reforming, and subsequently process of syngas conversion into CH_3_OH by Fischer‐Tropsch synthesis.^[^ ^8^, ^9^ ^]^ Nonetheless, the requirement to separate reaction steps and maintain high operating temperatures (*c.a*. over 800 °C) poses significant challenges to sustainable development.^[^ ^1^, ^10^ ^]^ With the advancement of the chemical industry, selective oxidation technology provides a promising method for the direct conversion of CH_4_ to CH_3_OH.^[^ ^6^, ^11^ ^]^ However, as the most basic saturated hydrocarbons, each methane (CH_4_) molecule consists of four C─H covalent bonds, making up ≈74% of the ground‐state description.^[^ ^12^ ^]^ This results in an exceedingly high bond energy of 434 kJ/mol. Coupled with its low‐lying highest occupied molecular orbital (HOMO) and high‐lying lowest unoccupied molecular orbital (LUMO), the stability of the CH_4_ molecule is remarkably high.^[^ ^1^ ^]^ Conversely, as the target products, high‐value‐added chemicals like methanol (CH_3_OH) are more reactive than CH_4_.^[^ ^5^ ^]^ Once the first C─H bond in a CH_4_ molecule dissociates, a chain reaction of dehydrogenation occurs from a thermodynamic perspective, leading to the production of thermodynamically favored CO and carbon dioxide (CO_2_), rather than the desired CH_3_OH.^[^ ^13^ ^]^ Additionally, it is reported that the energy barrier for the first C─H bond dissociation is higher than that of subsequent C─H bond dissociation, indicating a propensity for ongoing dehydrogenation in the CH_4_ conversion process from dynamics.^[^ ^14^ ^]^ For example, Roy et al. simulated the dissociation process of CH_4_ molecule on Ni_4_ cluster. It is found that the energy barrier (E_a_) for the initial C─H bond dissociation is 1.28 eV, which is considerably higher than that for subsequent dissociation (CH_3_ + H → CH_2_ + 2H, E_a_ = 0.42 eV; CH_2_ + 2H → CH + 3H, E_a_ = 0.29 eV).^[^ ^14^ ^]^ In essence, the peroxidation of target products is an inevitable process that poses one of the most significant challenges to be addressed in the direct conversion of CH_4_ into CH_3_OH.

In 1993, Periana and coworkers successfully produced the CH_3_OH products in the strongly acidic media of oleum.^[^ ^15^ ^]^ They discovered that the presence of oleum facilitates the formation of CH_3_OSO_3_H intermediates (CH_4_ + 2H_2_SO_4_ → CH_3_OSO_3_H + 2H_2_O + SO_2_). This process effectively inhibits the continuous dehydrogenation reaction, thereby promoting the production of CH_3_OH. Subsequently, in an effort to minimize environmental harm, researchers have strived to implement this reaction in aqueous solutions, thereby avoiding the use of highly acidic media.^[^ ^6^, ^11^ ^]^ For instance, Xiao et al. have designed the AuPd alloy nanoparticles embedded in aluminosilicate zeolite crystals, which can prevent the diffusion of hydrogen peroxide (H_2_O_2_; the oxidant in the reaction process) away from the reactive sites.^[^ ^11^ ^]^ Thanks to the increased concentration of H_2_O_2_, the catalyst reveals high methanol selectivity of 92% under 30 bar in the aqueous solution. It's important to note that the harsh operational conditions of strong acidic media or high pressure are crucial for avoiding the peroxidation of CH_3_OH products, which culminates in significantly environmental and economic sustainability challenges. Toward this end, there is a pressing need to explore alternate methods to achieve the direct conversion of CH_4_ to CH_3_OH under mild conditions.

Photocatalysis is a technology capable of overcoming thermodynamic limitations to selectively convert inert molecules into target products under ambient conditions (1 bar and approximately room temperature).^[^ ^16^ ^]^ This method of catalysis distinguishes itself from other methods (*c.a*. thermalcatalysis) by directly harnessing solar light, a clean, pollution‐free, and sustainable energy source, which negates the need for high temperature and pressure typically generated by burning fossil fuels.^[^ ^17^ ^]^ Furthermore, the direct conversion of CH_4_ into CH_3_OH would boost the energy density from a modest ≈36 kJ l^−1^ to a significant ≈17 MJ l^−1^.^[^ ^3^, ^4^ ^]^ In other words, if we could harness solar energy and store it successfully in the form of CH_3_OH, we could significantly augment the energy supply. In 2014, Murcia‐López. and coworkers conducted a study using bismuth‐based photocatalysts (Bi_2_WO_6_ and BiVO_4_) to directly convert CH_4_ into CH_3_OH under light irradiation.^[^ ^18^ ^]^ Then, in 2018, Junwang Tang and Ding Ma et al. achieved a high CH_4_ conversion rate (≈15%) with nearly 90% CH_3_OH selectivity by using H_2_O_2_ as the oxidant on FeO_x_/TiO_2_ photocatalyst.^[^ ^19^ ^]^ These findings suggest a promising future for photocatalysis in the highly selective conversion of CH_4_ into CH_3_OH through the effective inhibition of product peroxidation.

It is noted that various reviews summarizing the current research advancements in CH_4_ photooxidation have been already published.^[^ ^1^, ^5^, ^20^ ^]^ For example, Junwang Tang et al. penned an extensive overview of the development of photocatalysts in CH_4_ photooxidation.^[^ ^20^ ^]^ Zhiyong Tang and coworkers reviewed the mechanism of CH_4_ activation during the photocatalytic CH_4_ conversion.^[^ ^20^ ^]^ However, a minimal number of published reviews comprehensively summarize the mechanism behind inhibiting product peroxidation. This crucial aspect, despite its importance, generally remains under‐examined and insufficiently discussed, thereby leaving a noticeable void in the body of existing reviews. Herein, we aim to provide a comprehensive summary of the mechanisms and regulation methods used to inhibit product peroxidation in methane photooxidation. Based on the published works (cf. **Table** **1**), we categorize the methanol peroxidation inhibition mechanism into two primary types based on the nature of the active species: radical mechanism and active site mechanism. The radical mechanism involves the inhibition of methanol peroxidation through precise regulation of typical active radicals (hydroxyl radicals and hydroperoxyl radicals) generated during CH_4_ photooxidation. In contrast, the active site mechanism including methanol desorption and methanol regeneration mechanisms, relies on the active site regulation to suppress its peroxidation. Finally, we shed light on the challenges and prospects relating to the inhibition of the methanol peroxidation mechanism. Through this review, our goal is to deepen the mechanistic understanding of the methane photooxidation process and provide valuable guidance for the design of efficient photocatalysts with high selectivity.

**Table 1:** Representative works on photooxidation of methane to methanol.

Oxidants	Photocatalysts	Reaction conditions	Products	Selectivity of CH_3_OH	Mechanism	Reference
H_2_O_2_	pristine m‐WO_3_	≈1 bar, 25 °C, 300 W Xe lamp (420 nm < λ < 780 nm), 20 mg, 20% CH_4_/Ar	CH_3_OH CO_2_ C_2_H_6_	≈25%	radical mechanism	[#0021]
H_2_O_2_	1.98% FeOOH/m‐WO_3_	≈1 bar, 25 °C, 300 W, Xe lamp (420 nm < λ < 780 nm) 20 mg, 20% CH_4_/Ar	CH_3_OH CO_2_ C_2_H_6_	≈91.0%	radical mechanism	[#0021]
H_2_O_2_	0.33_metal_wt.% FeO_x_/TiO_2_	≈1 bar, 25 °C, Xe lamp (300 W, 710 nm), 20 mg, 20% CH_4_/Ar	CH_3_OH CO_2_ CH_3_CH_2_OH	>90.0%	radical mechanism	[#0019]
H_2_O_2_	0.1 wt.% Au_1_/WO_3_	≈20 bar, room temperature, 300 W Xe lamp (λ ≥ 420 nm), 20 mg, 20% CH_4_/Ar	CH_3_OH HCHO HCOOH CH_3_CHO	≈75.0%	radical mechanism	[#0022]
H_2_O_2_	0.15 wt.% Pd1/2DT	20 bar, 25 °C, 300 W Xe lamp (λ > 420 nm), CH_4_: 99.999%, 20 mg	CH_3_OH HCHO	≈94.6%	radical mechanism	[#0023]
H_2_O_2_	g‐CN	30 bar, 35 °C, 300 W Xe lamp (AM 1.5), CH_4_: 30 bar, 20 mg	CH_3_OH HCOOH CO CO_2_	≈63%	radical mechanism	[#0024]
H_2_O_2_	TiO_2_, Fe^2+^	30 bar, 35 °C, 300 W Xe lamp (AM 1.5), CH_4_: 30 bar, 20 mg	CH_3_OH HCOOH CH_3_CHO	≈84%	radical mechanism	[#0025]
H_2_O_2_	FeOOH/Li_0.1_WO_3_	20 bar, 30 °C, Xe lamp (λ ≥ 800 nm), CH_4_: 20 bar, 20 mg	CH_3_OH HCOOH CH_3_CHO	≈86%	radical mechanism	[#0026]
H_2_O_2_	RhB/TiO_2_	20 bar, 25 °C, Monochromic green light (550 nm), CH_4_: 0.034 mmol mL‐1, 20 mg	CH_3_OH CO	≈94%	radical mechanism	[#0027]
O_2_	0.1 wt.% Pd/ZnO	21 bar, 25 °C, Xe lamp (300 nm ≤ λ ≤ 500 nm), CH_4_: 20 bar, 10 mg	CH_3_OH CH_3_OOH HCHO CO CO_2_	≈26.2%	radical mechanism	[#0028]
O_2_	0.1 wt.% Au/ZnO	21 bar, 25 °C, Xe lamp (300 nm ≤ λ ≤ 500 nm), CH_4_: 20 bar, 10 mg	CH_3_OH CH_3_OOH HCHO CO CO_2_	≈15.7%	radical mechanism	[#0028]
O_2_	0.1 wt.% Pt/ZnO	21 bar, 25°C, Xe lamp (300 nm ≤ λ ≤ 500 nm), CH_4_: 20 bar, 10 mg	CH_3_OH CH_3_OOH HCHO CO CO_2_	≈19.1%	radical mechanism	[#0028]
O_2_	0.1 wt.% Ag/ZnO	21 bar, 25 °C, Xe lamp (300 nm ≤ λ ≤ 500 nm), CH_4_: 20 bar, 10 mg	CH_3_OH CH_3_OOH HCHO CO CO_2_	≈5.0%	radical mechanism	[#0028]
O_2_	Au_0.3_/ZnO	20 bar, 30 °C, Xe lamp (full light spectrum), CH_4_: 15 bar, 10 mg	CH_3_OH CH_3_OOH CO_2_	≈82.7%	radical mechanism	[#0029]
O_2_	q‐BiVO_4_	20 bar, 25 °C, Xe lamp (400 nm ≤ λ ≤ 700 nm), CH_4_: 10 bar, 10 mg	CH_3_OH HCHO CO_2_	≈96.6%	radical mechanism	[#0030]
O_2_	Au_1_/BP	33 bar, 90 °C, Xe lamp (full spectrum light), CH_4_: 30 bar, 200 mg	CH_3_OH	≈99.0%	radical mechanism	[#0031]
O_2_	PMOF‐RuFe(OH)	≈1 bar, 25 °C, visible light, CH_4_: 10 bar, 10 mg	CH_3_OH	≈100%	radical mechanism	[#0003]
H_2_O	Ga_2_O_3_	≈1 bar, 40 °C, 300 W Xe lamp, 1.25% CH_4_, 10 mg	CH_3_OH HCHO CO CO_2_	≈87.0%	radical mechanism	[#0032]
H_2_O	Cu‐0.5/PCN	≈1 bar, room temperature, 500 W Xe lamp, 10% CH_4_, 20 mg,	CH_3_OH C_2_H_5_OH	‐	radical mechanism	[#0033]
H_2_O	3.6‐Co‐SrTiO_3_	≈1 bar, 80 °C, Xe lamp, 10% CH_4_, 50 mg	CH_3_OH CO	≈98.7%	radical mechanism	[#0034]
H_2_O	WO_3_	≈1 bar, 55 °C, mercury lamp, UVC‐ visible light, CH_4_: 4.5 mL min^−1^, 0.3 g	CH_3_OH CO_2_ C_2_H_6_	≈38.2%	radical mechanism	[#0035]
H_2_O	La/WO_3_	≈1 bar, 55 °C, mercury lamp, UVC‐ visible light, CH_4_: 4.5 mL min^−1^, 0.3 g	CH_3_OH CO_2_ C_2_H_6_	≈47.0%	radical mechanism	[#0036]
H_2_O	BiVO_4_	≈1 bar, 55 °C, 450 W mercury lamp, UVC‐visible light, CH_4_: 20% CH_4_/He, 1 g L^−1^	CH_3_OH CO_2_ C_2_H_6_	≈48%	radical mechanism	[#0018]
O_2_	TiO_2{001}_	21 bar, 30 °C, 300 W Xe lamp, CH_4_: 20 bar, 10 mg	CH_3_OH HCHO CO CO_2_	≈41.9%	active site mechanism	[#0037]
O_2_	3.2% Ag/TiO_2{001}_	21 bar, 30 °C, 300 W Xe lamp, CH_4_: 20 bar, 10 mg	CH_3_OH HCHO CO CO_2_	≈79.4%	active site mechanism	[#0037]
O_2_	2.1% Ag/TiO_2{101}_	21 bar, 30 °C, 300 W Xe lamp, CH_4_: 20 bar, 10 mg	CH_3_OH HCHO CO	≈68.7%	active site mechanism	[#0037]
O_2_	BN	≈1 bar, 60 °C, 300 W Xe lamp, 1.25% CH_4_, 20 mg	CH_3_OH HCHO	(CH_3_OH+HCHO) ≈100%	active site mechanism	[#0038]

## 2 Radical Mechanism

It is well known that active radicals play a vital role in the photocatalytic conversion of CH_4_ into CH_3_OH. In general, the C─H bonds in the CH_4_ molecule can be attacked, abstracting a hydrogen atom by active radicals (CH_4_ + R· → ·CH_3_ + RH; where R represents active radicals). The generated methyl radicals (·CH_3_) can then directly combine with the active radicals and further convert into CH_3_OH. However, active radicals typically possess strong oxidation ability, which enables them to further attack the C─H bonds in CH_3_OH products, resulting in peroxidation. Therefore, controlling active radicals is critical for inhibiting the peroxidation of CH_3_OH. In this section, we summarize the typical reactive radicals generated during CH_4_ photooxidation to CH_3_OH and introduce their influence on the peroxidation of desired products. Furthermore, strategies to prevent peroxidation of desired products by regulating the generation of active radicals are reviewed in detail.

### 2.1 OH Mechanism

During the process of CH_4_ photooxidation, hydroxyl radical (·OH) is one of the most common active radicals generated in aqueous solution. As mentioned above, ·OH facilitates the activation of C─H bonds in CH_4_ molecules, but also causes undesired peroxidation of products. Previous studies suggest that the high concentration of ·OH would dramatically accelerate the peroxidation of CH_3_OH.^[^ ^19^, ^21^, ^22^, ^23^, ^24^, ^25^, ^26^, ^27^, ^28^, ^39^ ^]^ However, if the reaction system blindly lowers the concentration of ·OH, the activation of CH_4_ and the cleavage of its first C─H bond would be significantly inhibited. Therefore, to achieve the effective conversion of CH_4_ into CH_3_OH, it is crucial to strike a balance between optimizing CH_4_ activation and preventing CH_3_OH peroxidation by precisely regulating the concentration of ·OH. According to the generated source of ·OH, methods to regulate its concentration can be categorized into three main approaches.

#### 2.1.1 OH Generated by H_2_O_2_

As summarized in **Table** **2**, the ·OH can be generated by decomposing H_2_O_2_ (H_2_O_2_ + H^+^ + e^−^ → ·OH + H_2_O; H_2_O_2_/ ·OH = 1.14 V versus normal hydrogen electrode (NHE)). Consequently, a typical method to regulate the concentration of ·OH is optimizing the amount of supplemented H_2_O_2_.^[^ ^19^, ^21^ ^]^ For example, Junwang Tang and Ding Ma et al. found that without the introduction of H_2_O_2_ during the reaction process of CH_4_ conversion into CH_3_OH, no CH_4_ was converted.^[^ ^19^ ^]^ Elevating H_2_O_2_ increased CH_4_ conversion, but reduced CH_3_OH selectivity. The optimal ratio of H_2_O_2_ to CH_4_ was 0.11 when FeO_x_/TiO_2_ was used as the photocatalyst. Furthermore, introducing cocatalysts to regulate the conversion of H_2_O_2_ into ·OH has been recognized as an effective strategy to enhance the selectivity of CH_3_OH when H_2_O_2_ is used as the oxidant.^[^ ^22^, ^23^, ^25^, ^26^ ^]^ For instance, Wang et al. employed the Fenton reaction to manage the transformation of H_2_O_2_. They discovered that with the addition of Fe^2+^, the selectivity of CH_3_OH over the TiO_2_ photocatalyst could reach 83%, attributed to the enhanced generation of ·OH from the Fenton reaction (Fe^2+^ + H_2_O_2_ → Fe^3+^ + ·OH + OH^−^).^[^ ^25^ ^]^

**Table 2:** The typical generation pathways of ·OH.

Entry	Generation Pathway	Redox Potential (V vs NHE)
1	H_2_O_2_ + H^+^ + e^−^ → ·OH + H_2_O	H_2_O_2_/·OH = 1.14
2	2H_2_O + 2h^+^ → H_2_O_2_ + 2H^+^	H_2_O/H_2_O_2_ = 1.76
	H_2_O_2_ + H^+^ + e^−^ → ·OH + H_2_O	H_2_O_2_/·OH = 1.14
3	H_2_O + h^+^ → ·OH + H^+^	H_2_O/·OH = 2.38
4	O_2_ + 2H^+^ + 2e^−^ → H_2_O_2_	O_2_/H_2_O_2_ = 0.70
	H_2_O_2_ + H^+^ + e^−^ → ·OH + H_2_O	H_2_O_2_/·OH = 1.14

#### 2.1.2 OH Generated by H_2_O Oxidation

**Figure 1:**
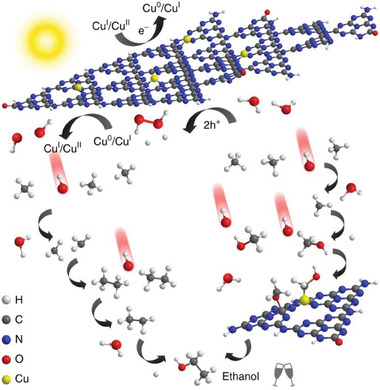
The hypothetic mechanism for photocatalytic anaerobic methane conversion over Cu‐0.5/PCN. Reproduced with permission.^[^ ^33^ ^]^ Copyright 2019, Springer Nature.

Apart from the decomposition of H_2_O_2_, ·OH can also be produced via water (H_2_O) oxidation. However, as illustrated in **Table** **2**, there are two distinct pathways (direct pathway and indirect pathway) for converting H_2_O to ·OH, leading to different regulation strategies. With the indirect method, adsorbed H_2_O molecules initially transform into H_2_O_2_ (2H_2_O + 2h^+^ → H_2_O_2_ + 2H^+^; H_2_O/H_2_O_2_ = 1.76 V vs NHE;) and are then decomposed into ·OH (H_2_O_2_ + H^+^ + e^−^ → ·OH + H_2_O; H_2_O_2_/·OH = 1.14 V vs NHE). This two‐step conversion process can lead to a decrease in ·OH concentration, particularly as H_2_O_2_ intermediates can easily diffuse away from the active sites, disrupting subsequent reactions. Therefore, mitigating the diffusion of H_2_O_2_ becomes critical in ·OH concentration regulation. As mentioned earlier in Introduction, H_2_O_2_ diffusion can be markedly constrained by designing a molecular fence.^[^ ^11^, ^33^ ^]^ For instance, covering active sites with a hydrophobic layer helps prevent H_2_O_2_ diffusion.^[^ ^11^ ^]^ In 2019, Wang and coworkers pioneered a novel method to control ·OH concentration by dynamically modulating the copper (Cu) valence state.^[^ ^33^ ^]^ As depicted in **Figure** **1**, they demonstrated that in situ decomposition of H_2_O_2_, generated from H_2_O conversion, occurred with the help of Cu, significantly preventing H_2_O_2_ diffusion.

**Figure 2:**
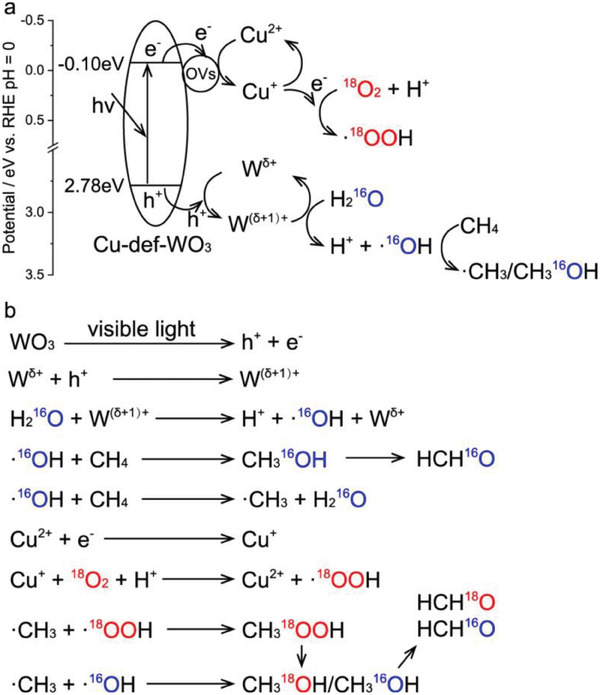
Schematic of charge transfer steps during selective CH_4_ oxidation over Cu_0.029_‐def‐WO_3_. Reproduced with permission.^[^ ^40^ ^]^ Copyright 2023, Springer Nature.

In contrast to the indirect method, due to the direct conversion of H_2_O into ·OH (H_2_O + h^+^ → ·OH + H^+^, ·OH/H_2_O = 2.38 V versus NHE), optimizing ·OH concentration is typically achieved through regulating H_2_O activation. A prevalent method to maneuver the H_2_O activation is to introduce the active sites that could trap photo‐generated holes, consequently activating the H_2_O molecules and directly converting them into ·OH.^[^ ^40^ ^]^ For example, Junwang Tang and Zhengxiao Guo et al. found that W^δ+^ species in defective tungsten oxide nanocrystals (WO_3‐x_) operated as hole acceptors, offering enhanced H_2_O adsorption and activation to generate ·OH (cf. **Figure** **2**).^[^ ^40^ ^]^ A single Cu atom synergistic effect enables nearly 100% formaldehyde (HCHO) selectivity without any generation of peroxidation products.

However, the redox potential of direct H_2_O conversion into ·OH is *c.a*. 2.38 V versus NHE, implicating numerous photo‐generated holes with powerful oxidation capability generated by photocatalysts under light irradiation. Photo‐generated holes, rather than the ·OH, tend to induce the activation of CH_4_ molecules and their subsequent transformation into ·CH_3_. In this case, if the reaction systems exist a high concentration of ·OH, the generated CH_3_OH will further transform into peroxidation products. Consequently, to avoid CH_3_OH peroxidation, it is often necessary to reduce the ·OH concentration by limiting the direct conversion of H_2_O into ·OH. This reduction is typically achieved through defect engineering. By fabricating a defect on the surface of the photocatalyst, this defect will become a site to trap photo‐generated holes but not to adsorb H_2_O, thereby the formation of ·OH can be inhibited to prevent CH_3_OH peroxidation.^[^ ^37^ ^]^ For example, as observed by Feng and Ye et al., creating oxygen vacancies on the {001} facet of anatase titanium dioxide (TiO_2_), instead of the {101} facet, could control the transfer of photo‐generated holes, increasing CH_3_OH selectivity from 64.6% to 79.4%.^[^ ^37^ ^]^

**Figure 3:**
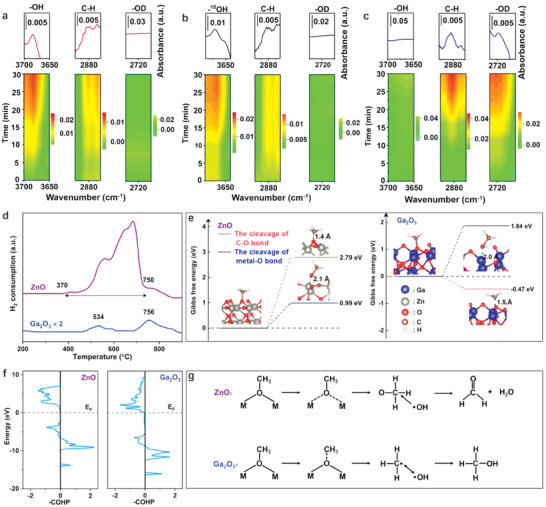
In situ DRIFTS spectra of generated CH_3_OH‐related species on Ga_2_O_3_ under light irradiation with the introduction of mixtures of CH_4_ and H_2_O a), H_2_^18^O b), or D_2_O c) vapor. These data are processed for smoothing. d) H_2_‐TPR spectra; e) Gibbs free‐energy profiles corresponding to the cleavage of different chemical bonds in the CH_3_O* intermediates; f) pCOHP curves of selective C−O bonds in the CH_3_O* intermediates; and the g) proposed reaction pathways. Reproduced with permission.^[^ ^32^ ^]^ Copyright 2023, American Chemical Society.

While limiting the direct conversion of H_2_O into ·OH can alleviate CH_3_OH peroxidation to some extent, the generated ·OH is required to contribute to subsequent CH_3_OH formation (·CH_3_ + ·OH → CH_3_OH). Hence, reducing the ·OH concentration thoughtlessly would curtail CH_4_ transformation into CH_3_OH. To address this, we have proposed a new method to control the CH_4_ conversion pathway by selectively cleaving the chemical bonds in key intermediates, significantly reducing CH_3_OH peroxidation.^[^ ^32^, ^41^ ^]^ As illustrated in **Figure** **3**, we found that the cleavage of different chemical bonds (C─O bonds or metal─O bonds) in CH_3_O* intermediates could considerably affect the peroxidation process of CH_3_OH through combined density functional theory calculations and in situ infrared spectroscopy based on ^18^O isotope labeling. Specifically, timely C─O bond cleavage in CH_3_O* intermediates leads to the generated ·CH_3_ rapidly combining with ·OH to form CH_3_OH. Conversely, preferred metal‐O bonds cleavage results in the produced ·OH attacking C─H bonds in ·CH_3_O, prompting continuous dehydrogenation to yield peroxidation products of CO_2_. Based on these findings, we modulated lattice oxygen mobility to achieve precise electron injection into the antibonding orbitals of designated chemical bonds in CH_3_O* intermediates to regulate the selective cleavage of C─O bonds. Consequently, under room temperature and atmospheric pressure without extra oxidants, the CH_3_OH product selectivity increased to ≈87.0% with a CH_3_OH yield of 325.4 µmol g^−1^ h^−1^ ‐a superior result compared to reported studies (reaction pressure: <20 bar; in April 2023).

#### 2.1.3 OH Generated by O_2_ Reduction

Additionally, another pathway to generate ·OH is through oxygen (O_2_) reduction. Conventional processes in thermalcatalysis first combine hydrogen (H_2_) with O_2_ to produce H_2_O_2_, and then decomposes into ·OH.^[^ ^11^ ^]^ Similarly, the photocatalysis process for O_2_ conversion into ·OH requires O_2_ molecules to combine with a proton‐coupled photo‐generated electron to produce H_2_O_2_ (O_2_ + 2H^+^ + 2e^−^ → H_2_O_2_; O_2_/H_2_O_2_ = 0.70 V vs NHE). H_2_O_2_ then decomposes into ·OH (H_2_O_2_ + H^+^ + e^−^ → ·OH + H_2_O; H_2_O_2_/·OH = 1.14 V vs NHE). However, the presence of O_2_ accelerates CH_3_OH peroxidation due to effective C─H bond activation from reactive thermodynamics.^[^ ^13^, ^38^ ^]^ Hence, in such situations, it is essential to maintain the ·OH concentration below thresholds observed in systems without O_2_. Cocatalysts are commonly introduced onto the photocatalyst surface to reduce the ·OH concentration.^[^ ^39^, ^42^ ^]^ As an example, Meng, Wang and Ye et al. found that when cobalt oxide nanoclusters (CoO_x_) were introduced on noble metals (Au, Pd, Ag, and Pt) modified titanium dioxide photocatalysts (CoO_x_‐NM/TiO_2_), the photo‐generated holes would transfer from TiO_2_ to CoO_x_, significantly suppressing ·OH generation.^[^ ^39^ ^]^ This modification led to a decrease in peroxidation product (CO_2_) selectivity to ≈5% owing to a significant ·OH concentration reduction. Subsequently, the exposed crystal facets of TiO_2_ are adjusted to minimize the generation of ·OH, thereby inhibiting the peroxidation of CH_3_OH. It was observed that TiO_2_ with a dominant {001} facet exhibited ≈80% selectivity for CH_3_OH.^[^ ^37^ ^]^

It could be concluded that when the peroxidation of CH_3_OH follows by the ·OH mechanism, the key point is to accurately control the concentration of ·OH. In this case, if the ·OH is generated by H_2_O_2_ decomposition, the optimal approach is to accurately tune the ratio of added H_2_O_2_ and CH_4_ reactants. For the ·OH generated by a two‐step H_2_O oxidation, preventing H_2_O_2_ diffusion from reactive sites by designing a molecular fence is crucial. Besides that, if the ·OH is generated via O_2_ reduction or H_2_O direct oxidation, the decrease of ·OH concentration is vital to prevent product peroxidation. In this case, controlling the photo‐generated hole transfer is an effective strategy through the fabrication of defects or the introduction of cocatalysts. Furthermore, the selective chemical bond cleavage in key intermediates also plays a significant role in inhibiting peroxidation products and can be achieved by modulating photo‐generated electron transfer. Therefore, when without introducing H_2_O_2_ as the oxidant, modulating the photo‐generated charge transfer is imperative to avoid the peroxidation of desired CH_3_OH products.

### 2.2 OOH Mechanism

**Figure 4:**
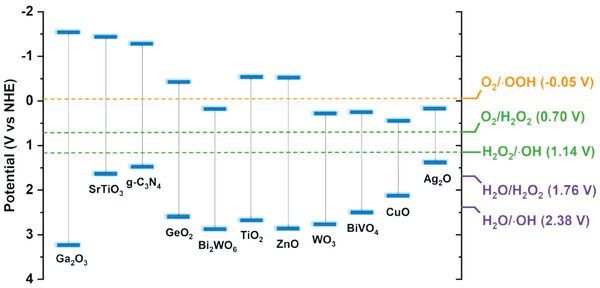
Illustration of the band structures of common‐used semiconductors in CH_4_ photooxidation and the redox potentials of diverse oxidants.

In addition to the ·OH mechanism, the hydroperoxyl radicals (·OOH) mechanism can also be utilized to prevent the peroxidation of CH_3_OH. In 2019, Ye et al. reported the ·OOH mechanism during the process of CH_4_ photooxidation by using ZnO loaded with noble‐metal cocatalysts (Pt, Pd, Au or Ag) as the photocatalysts.^[^ ^28^ ^]^ One advantage of this mechanism is that it only requires molecular oxygen as the reactant under light irradiation to drive the CH_4_ photooxidation. More importantly, compared with ·OH, ·OOH is more mildly oxidative, which inhibits the formation of peroxidation products like CO and CO_2_.^[^ ^30^ ^]^ However, as presented in **Figure** **4**, when the band edge of photocatalysts used in CH_4_ photooxidation meets the redox potential for converting O_2_ into ·OOH (O_2_ + H^+^ + e^−^ → ·OOH; O_2_/·OOH = −0.05 V vs NHE), the reaction for the conversion of O_2_ into ·OH could also take place (O_2_ + 2H^+^ + 2e^−^ → H_2_O_2_, H_2_O_2_ + H^+^ + e^−^ → ·OH + H_2_O; O_2_/H_2_O_2_ = 0.70 V vs NHE, H_2_O_2_/·OH = 1.14 V vs NHE). In other words, ·OOH formation generally accompanies the conversion process of O_2_ conversion into ·OH. In this case, although the ·OOH reveals a mild oxidation ability, the presence of ·OH possessing a strong oxidation can still lead to the generation of unavoidable thermodynamic‐favored peroxidation products. Therefore, considerable efforts have focused on promoting ·OOH generation as well as inhibiting ·OH formation to prevent the peroxidation of CH_3_OH when the CH_4_ photooxidation follows by the ·OOH mechanism.

**Figure 5:**
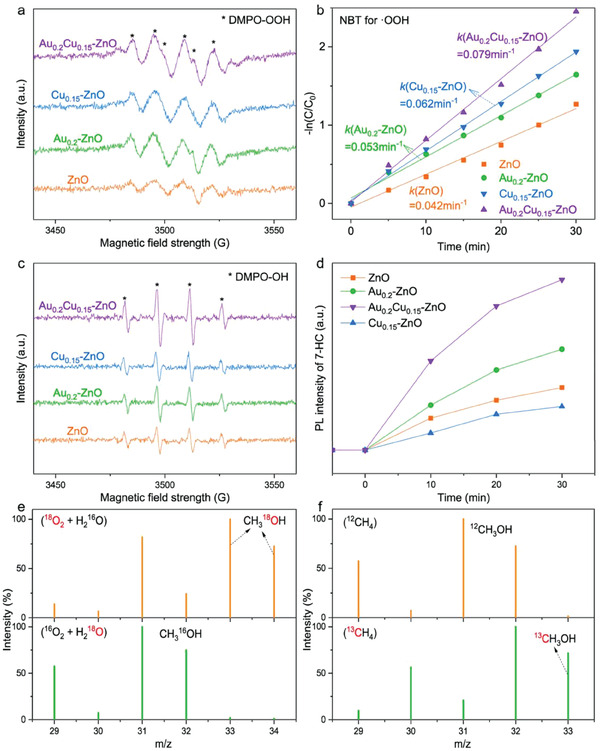
In situ EPR spectra of a) DMPO‐OOH and c) DMPO‐OH for monitoring the generation of· OH and ·OOH active species over different photocatalysts. b) The kinetic constant of photodegradation of NBT for ·OOH radical detection over different photocatalysts. d) Timedependent PL spectra of the produced 7‐hydroxycoumarin for ·OH radical detection over different photocatalysts. GC‐MS results of the isotope labeling experiments in the presence of e) ^16^O_2_ + H_2_^18^O or ^18^O_2_ + H_2_^16^O and f) 5 bar ^13^CH_4_ or 5 bar ^12^CH_4_. Reproduced with permission.^[^ ^42^ ^]^ Copyright 2022, American Chemical Society.

As summarized above, introducing cocatalysts is a common strategy to inhibit ·OH formation by modulating photo‐generated charge transfer. In order to promote ·OOH generation, introducing Cu species is touted as one of the most effective methods for achieving a highly selective conversion of O_2_ into ·OOH.^[^ ^28^, ^39^, ^42^ ^]^ On one hand, the reduction of Cu^2+^ to Cu^+^ provides an electron to participate in the reaction process of O_2_ conversion into ·OOH. On the other hand, Cu species could serve as the photo‐generated electron mediators under light irradiation, supplying ample electrons for ·OOH formation.^[^ ^40^, ^42^ ^]^ As an example, Junwang Tang and coworkers enhanced the selectivity of C1 oxygenates (CH_3_OH, CH_3_OOH and HCHO) to nearly 100% by introducing Cu species on Au‐loaded ZnO photocatalysts (cf. **Figure** **5**).^[^ ^40^ ^]^ Additionally, manipulating the size of nanomaterials could also enhance the generation of ·OOH, thereby increasing the selectivity of CH_3_OH products.^[^ ^30^, ^43^ ^]^ For instance, Zhiyong Tang and coworkers found that when bismuth vanadate nanomaterials (BiVO_4_) were downsized to quantum size, the yield of target CH_3_OH products increased by a four‐factor.^[^ ^30^ ^]^ After that, they optimized the size of Au active sites (single atoms or nanoparticles) on Au‐loaded indium oxide (Au/In_2_O_3_) to control the types of generated active radicals.^[^ ^43^ ^]^ It is found that when the size of Au nanostructures changed from single atoms to nanoparticles, the main type of active radicals switched from ·OH to ·OOH, leading to a significant increase in CH_3_OH products by 89.4%.

In brief, the ·OOH mechanism opens a new pathway to inhibit CH_3_OH peroxidation because of its mild oxidation ability. However, owing to the co‐existence of ·OOH and ·OH (with strong oxidation capacity), unavoidable CH_3_OH peroxidation can still occur when CH_4_ photooxidation follows by the ·OOH mechanism. To address this issue, significant efforts are being made in introducing Cu species and adjusting the size of nanomaterials or active sites to inhibit ·OH formation while promoting ·OOH generation, which suppresses the peroxidation of CH_3_OH. It is important to note that the generation of active radicals is closely tied to the photo‐generated charges and the relationship warrants further exploration.

## 3 Active Site Mechanism

Beyond the radical mechanism, the active site mechanism assumes a crucial role in suppressing the peroxidation of CH_3_OH in the process of CH_4_ photooxidation. Employing the active site mechanism could promote the desorption of produced CH_3_OH and make it diffuse away from the active sites, thus suppressing its peroxidation. Additionally, the photoreduction of peroxide products to regenerate CH_3_OH through active site regulation presents an innovative pathway for improving the selectivity of CH_3_OH products. In this section, we delve into various active site mechanisms, including the methanol desorption mechanism and methanol regeneration mechanism. The strategies to prevent CH_3_OH peroxidation via the active site mechanism are also comprehensively summarized.

### 3.1 Methanol Desorption Mechanism

**Figure 6:**
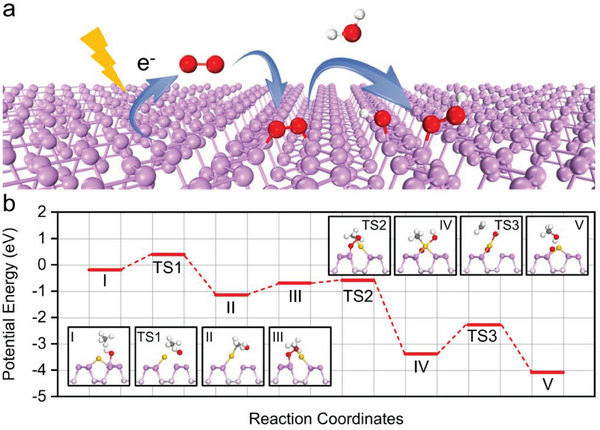
DFT studies. a) Schematic illustration of oxygen activation on BP nanosheets. b) Reaction path for partial oxidation of methane over Au_1_/BP nanosheets under light irradiation. The inset images show the side views of the configurations. Yellow, violet, pink, red, gray, and white spheres represent Au, surface P, subsurface P, O, C, and H atoms, respectively. Reproduced with permission.^[^ ^31^ ^]^ Copyright 2021, Springer Nature.

Among the array of active site mechanisms, the methanol desorption mechanism is the most frequently employed. This mechanism promotes the desorption of CH_3_OH from the active sites, facilitated by the reaction media or the reconstruction of active sites, thus inhibiting further oxidation. For the regulation of reaction media, previous studies have indicated that a strongly acidic reaction media could enhance the selectivity of CH_3_OH.^[^ ^44^ ^]^ When replacing the strongly acidic reaction media with a mildly aqueous solution to reduce the damage to the environment, it is found that aqueous solution could also facilitate the effective desorption of CH_3_OH.^[^ ^4^, ^31^, ^45^ ^]^ For instance, a study by Li and Zeng et al. found that the substitution of water with acetonitrile as the reaction media under standard conditions resulted in no detected products over single‐atom gold on black phosphorus nanosheets (Au_1_/BP) under light irradiation (cf. **Figure** **6**).^[^ ^31^ ^]^ This implies that the presence of H_2_O solvent can promote the generation of CH_3_OH products. A similar observation was made by Jose A. Rodriguez and coworkers.^[^ ^3^ ^]^ They found that when a sufficient amount of water molecules was incorporated into the reaction systems, the chemical bonds between methyl species and the active sites of Ni particles were relatively easy to dissociate, resulting in an enhanced product selectivity.

Another efficient approach to inhibit CH_3_OH peroxidation by enhancing its desorption is the active site reconstruction. Similar to how we harness the reconstruction of active sites (e.g., Cu^2+^ → Cu^+^) to control the generation of active radicals, this method can also help to weaken the interaction between adsorbed CH_3_OH products and the active sites, leading to effective inhibition of CH_3_OH peroxidation.^[^ ^46^ ^]^ For instance, Sun and Xie et al. demonstrated that when Fe_2_O_3_ was introduced on the surface of ZnO photocatalyst, the electron structure of Fe sites alters and the accumulated charge strengthens the polarity of the O─H bond in CH_3_OH.^[^ ^46^ ^]^ This simultaneously weakens the O─metal bonds, thus enhancing CH_3_OH desorption and suppressing its peroxidation. In addition to introducing other active sites, our recent study found that adsorbed species of OH could reconstruct active sites.^[^ ^47^ ^]^ To be specific, after the formation of CH_3_OH products, the high spin magnetic moment of the active site of a single Ag atom can be reduced to nearly zero by the introduction of adsorbed OH species during the subsequent dehydrogenation process. This interrupts the spin channel for electron transfer between the adsorbed CH_3_OH and reactive sites, hence promoting its desorption to inhibit further peroxidation.

### 3.2 Methanol Regeneration Mechanism

**Figure 7:**
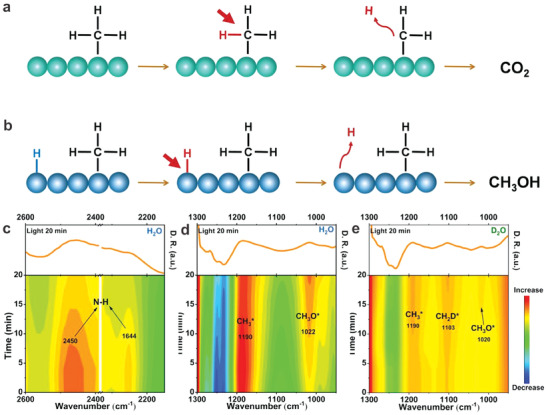
a) Typical CH_4_ photooxidation reaction process taking place on the surface of the catalyst, and b) CH_4_ photooxidation reaction process occurring on the BN surface. In situ DRIFTS spectra of BN under light irradiation with the introduction of CH_4_, O_2_, and H_2_O mixtures c) in the range of 2600–2100 cm^−1^, d) in the range of 1300–900 cm^−1^, and e) with the introduction of CH_4_, O_2_, and D_2_O mixtures in the range of 1300–900 cm^−1^. These data were smoothed during processing. Reproduced with permission.^[^ ^38^ ^]^ Copyright 2023, Wiley‐VCH.

The methanol desorption mechanism detailed above hinges on the enhancement of CH_3_OH desorption to obstruct its peroxidation. It's well established that the peroxidation of CH_3_OH is largely unavoidable owing to reaction thermodynamics. Since the continuous dehydrogenation process is inevitable, if we could devise a reaction pathway to regenerate CH_3_OH, it would effectively address this issue. With this in mind, we propose a novel method – proton rebound to regenerate CH_3_OH.^[^ ^38^ ^]^ As illustrated in **Figure** **7**, we designed N─H bonds to function as a hydrogen bonding trap to attract electrons on the BN surface. On one hand, this design ensures that N─H bonds on BN surfaces, rather than C─H bonds in target products are preferentially cleaved, thus significantly suppressing the dehydrogenation process. Of critical importance, this method facilitates the coupling of peroxidation products (HCHO) with the released protons from the cleavage of N─H bonds to regenerate CH_3_OH (HCHO + H^+^ + e^−^ → CH_3_OH). We have carried out the isotopic labeling experiment, for the first time, to track this process that HCHO pairs up with released protons, triggering the proton rebound to regenerate CH_3_OH. Consequently, in comparison to conventional photocatalysts (e.g., ZnO and P25) used in CH_4_ photooxidation, BN nanosheets demonstrated a high CH_4_ conversion rate of 8.5% and nearly 100% product selectivity to oxygenates under atmospheric pressure.

Different from the radical mechanism, the active site mechanism strives to intensify the desorption of CH_3_OH products from active sites, thereby fostering the inhibition of CH_3_OH peroxidation. Both reaction media and the reconstruction of active sites can effectively accomplish this aim. In addition, the methanol regeneration mechanism offers a new viewpoint for inhibiting the peroxidation of desirable products. This strategy warrants further exploration and implementation in other reaction systems.

## 4 Conclusion and Future Perspectives

Methane photooxidation into methanol offers a viable pathway to produce high‐value‐added chemicals and enable efficient solar energy storage. However, the low efficiency and product selectivity of methane photooxidation due to the facile peroxidation of target products hinders its commercial application. Various strategies have been employed to effectively inhibit product peroxidation, which can be classified into two major categories: radical mechanism and active site mechanism, depending on the nature of the active species.

At the crux of the radical mechanism is the precise control over the generation and transformation of radicals, facilitated by photo‐generated carrier transfer. On one hand, researchers aim to augment the effective injection of photo‐generated carriers into the sources of radical generation and key intermediates, promoting the generation of radicals with milder oxidation abilities (*c.a*. ·OOH). Commonly employed strategies include defect engineering, the size control of loading metals, etc. The primary role of these active sites, comprising both defects and metals, is to facilitate the adsorption capacity for O_2_ molecules and the activation of O─O bonds. To accomplish this goal, oxygen vacancies and noble metals with strong adsorption and activation capabilities for O_2_, are typically selected as active sites on photocatalyst surfaces. This selection is crucial for enabling the efficient transfer of electrons into O_2_ molecules and O_2_H* intermediates, thereby enhancing the generation of ·OOH. On the other hand, the formation of radicals with strong oxidation abilities (*c.a*. ·OH), needs to be restrained to prevent the peroxidation of the target products. One effective approach is to accurately optimize the quantity of added H_2_O_2_, which is frequently used as the source to generate ·OH. Another method involves severing the reaction pathway of ·OH generation through the modulation of photo‐generated carrier transfer, induced by cocatalysts. These cocatalysts provide active sites to trap photo‐generated carriers, thereby preventing the injection of them into the reactants to generate ·OH. All these strategies hinge on the regulation of ultrafast charge transfer dynamics in photocatalysts, an area that requires substantial future work.

Distinct from the radical mechanism, the primary focus of the active site mechanism is to inhibit the peroxidation of target products through active site regulation, promoting methanol desorption or regeneration. Methods to facilitate methanol desorption include modifying reaction media and reconstructing active sites. The former depends on enhancing the diffusion behavior of methanol away from active sites, induced by the reaction media (*c.a*. oleum, H_2_O). The latter relies on weakening the interaction between adsorbed methanol and the active sites, which is achieved by disrupting electron transfer through the dynamic reconstruction of active sites. Regarding the methanol regeneration mechanism, the central objective is to facilitate the effective combination of protons and peroxidation products to regenerate methanol, depending on the cooperation of multiple active sites. The exploration of synergistic effects between different active sites is an area that needs future research.

As we have outlined above, despite the significant advancements made in inhibiting product peroxidation in methane photooxidation, this field still faces several challenges that require ongoing investigation.

### 4.1 Development of Characterization Techniques

As previously summarized, both the radical mechanism and active site mechanism highly rely on the regulation of ultrafast charge transfer dynamics in photocatalysts, enabling effective inhibition of methanol peroxidation. However, the current use of time‐resolved spectroscopies is limited to monitoring the generation and transportation of photo‐generated carriers within photocatalysts. It poses difficulties in observing the involvement of photo‐generated carriers in the formation and conversion process of reaction intermediates, particularly active radicals. Therefore, there is an urgent need to employ advanced techniques such as synchrotron radiation photoionization mass spectrometry and design adaptive in situ reaction cells to track the generation and conversion process of reactive intermediates (e.g., active radicals) at the ultrafast timescale during the methane photooxidation reaction, aiming to achieve the precise regulation of ultrafast charge transfer dynamics to effectively inhibit the peroxidation of desired products.

### 4.2 Design of Photocatalysts

In addition to the development of characterization techniques, the design of photocatalysts plays a vital role in enhancing the efficiency and product selectivity of methane photooxidation. For the catalyst design, at least three challenges face this field.

First, it has been observed that the concentration of active radicals (e.g., ·OH) is crucial in the peroxidation of CH_3_OH. Various methods have been employed to adjust their concentration during the photooxidation of CH_4_ into CH_3_OH. However, regarding the regulation of ·OOH, in the existing literature, most of the studies have solely focused on the role of ·OOH in preventing the peroxidation of CH_3_OH, without discussing whether an excess of ·OOH might trigger CH_3_OH peroxidation. Future research is suggested to focus on investigating how an abundance of ·OOH affects product selectivity. Additionally, optimizing catalysts to convert O_2_ into ·OOH at an ideal concentration is vital for improving CH_3_OH selectivity. This approach will be key in precisely controlling the peroxidation of CH_3_OH during CH_4_ photooxidation.

Second, existing research has revealed that the active sites significantly influence the selective cleavage of chemical bonds in key intermediates, playing a crucial role in the selectivity of CH_3_OH. Various effective strategies, such as controlling lattice oxygen mobility, have been proposed for managing this selective cleavage of chemical bonds. Future research is recommended to further investigate the influence of the active sites (e.g., coordination environment) on the lattice oxygen mobility process. Such research could enable the precise engineering of catalysts that are more effective in suppressing CH_3_OH peroxidation.

Third, most of the current research focuses primarily on inhibiting CH_3_OH peroxidation, often overlooking the crucial aspect of effectively activating C─H bonds in CH_4_ molecules, a key step toward achieving the desired outcome. It's important to recognize that the design principles for photocatalysts aimed at CH_3_OH peroxidation inhibition differ markedly from those needed to activate C─H bonds in CH_4_ molecules. For instance, active sites that facilitate C─H bond activation in CH_4_ molecules typically require enhanced ability for molecule adsorption. Conversely, for preventing CH_3_OH peroxidation, active sites that favor molecule desorption may be more beneficial. Therefore, integrating multifunctional active sites into photocatalysts is essential to cater to these varied requirements. Furthermore, when multiple active sites are engineered on a photocatalyst surface, it is important to consider the interactions among these different active sites. Especially, under light irradiation, the transfer of photo‐induced carriers will occur between different activity sites, leading to the undesired recombination of photo‐induced carriers. Understanding and optimizing these interactions is vital to ensure the effective collaboration of different active sites in achieving the effective photooxidation of CH_4_ to CH_3_OH. Therefore, a thorough exploration of the synergistic effects between various active sites is essential.

### 4.3 Design of Reaction Systems

Currently, the majority of reported reaction systems are batch reaction systems, which present the challenges of inhibiting product peroxidation. Specifically, as the reaction time increases, the concentration of methane and oxidant reactants decreases while the concentration of methanol product increases. Failure to timely replenish reactants and separate products can lead to an unavoidable decrease in methanol selectivity. Hence, it is crucial to implement methane photooxidation in a flowing reaction system. Because of the complex methane photooxidation process, designing and utilizing flow reaction systems pose challenges. In the future, this field still necessitates considerable exploration.

## Conflict of Interest

The authors declare no conflict of interest.
